# Association between Body Mass Index and Physical Function among Endometrial Cancer Survivors

**DOI:** 10.1371/journal.pone.0160954

**Published:** 2016-08-16

**Authors:** Xiaochen Zhang, Justin C. Brown, Kathryn H. Schmitz

**Affiliations:** 1 Public Health Science, Penn State College of Medicine, Hershey, PA, United States of America; 2 Division of Population Sciences, Department of Medical Oncology, Dana-Farber Cancer Institute, Boston, MA, United States of America; Universidad Europea de Madrid, SPAIN

## Abstract

**Objectives:**

We sought to quantify the relationship between body mass index (BMI) and physical function among endometrial cancer survivors. Understanding this relationship would help healthcare providers target efforts to refer obese endometrial cancer survivors to weight loss and exercise interventions.

**Methods:**

We conducted a survey of 213 endometrial cancer survivors who received cancer care at an academic l health system between 2006 and 2010. Physical function subscale was quantified using physical functional component score from the SF-12 questionnaire. We compared physical function of endometrial cancer survivors to population-based age-standardized normative values.

**Results:**

Among the 213 patients, 16% were normal weight (BMI ≤25 kg/m^2^), and 52% were obese (≥30 kg/m^2^). Higher BMI categories were associated with lower physical function (*P*_trend_ = 0.003), as a continuous variable each 5kg/m^2^ higher BMI, physical function score was lower by 0.15 points (β = -0.15; *P* = 0.045). Compared to population-based age-standardized normative values, patients <75yrs reported lower physical function, whereas patients ≥75yrs reported better physical function. BMI was the only covariate associated with differences in physical function between survivors and age-standardized normative values (*P* = 0.039).

**Conclusions:**

Among endometrial cancer survivors, higher BMI is associated with lower physical function. Younger endometrial cancer survivors report lower physical function compared to age-standardized normative values. Healthcare providers should be aware that younger, obese endometrial cancer survivors may particularly benefit from interventions such as exercise and weight loss to increase or preserve physical function.

## Introduction

Endometrial cancer is the fourth most common gynecologic cancer among women in the United States [1], and often diagnosed at an early stage, with a five-year survival rate of 81.7% [2]. In 2015, there were over 600,000 endometrial cancer survivors in the United States [2]. As the length of survival after endometrial cancer continues to be extended, greater focus is being paid to the management of long-term health issues and preservation of quality of life [3]. Physical function is the ability to complete activities required for safe independent living. Quality of life may be compromised among individuals who are unable to independently complete essential activities of daily living [4]. Limitations in physical functioning predict clinical outcomes, including mortality and morbidity among the general population and cancer survivors [5–9].

Cancer and cancer treatment (surgery with or without adjuvant chemotherapy and/ or radiation), may contribute to physical dysfunction, musculoskeletal weakness, pain, fatigue, and depression among endometrial cancer patients, which may restrict or impair activities of daily living [10]. The prevalence of obesity is high among women with endometrial cancer, such that >70% are overweight or obese (body mass index [BMI] ≥25 kg/m^2^). Multiple studies have shown that community-dwelling elderly with higher BMI, particularly those who are obese (BMI ≥30 kg/m^2^), report lower physical function and more functional impairments than those who are of a healthy weight (BMI <25 kg/m^2^)[11–13]. Therefore, obesity and treatment-related sequelae may collectively result in poorer physical function and lower quality of life among endometrial cancer survivors.

Studies have shown that cancer survivors often report more functional limitations and health issues than non-cancer populations [14,15]. However, these studies have not investigated differences between the general population and endometrial cancer survivors exclusively. The purpose of this study was to assess the cross-sectional relationship between BMI and physical function in a hospital-based cohort of endometrial cancer survivors, and the differences in physical function between endometrial cancer survivors and the general population. This information may assist healthcare providers to identify patients who may be prone to report poor physical function and who may benefit from lifestyle interventions to maintain and improve their long-term functioning and quality of life.

## Materials and Methods

### Participants and Procedures

We conducted a cross-sectional survey of patients with endometrial cancer who received care at the University of Pennsylvania in Philadelphia, Pennsylvania[16–19]. Participants included women ≥20 years old, with a history of endometrial cancer. Potential eligible participants were identified using fellow surgical case logs from 2006–2010, and ICD-9 diagnosis codes 179.0 and 182.0–182.8, from 2006–2010. ICD-9 codes 179.0 and 182.0–182.8 are the primary codes used to classify cancers of the uterus (95% of them are endometrial cancer). Participants who met the study inclusion criteria were sent a letter from their oncologist explaining the purpose of the study. Participants who did not wish to participate were provided the option to decline participation within two weeks of receiving the letter from their oncologist. Those who did not decline participation were sent the study survey. After two weeks, a second survey was sent to those who did not reply to the first mailed survey. This protocol was approved by the University of Pennsylvania Institutional Review Board. Women who returned a completed survey were classified as having provided their informed consent.

### Physical Function

The Medical Outcomes Study 12-Item Short-Form Health Survey (SF-12) was used to assess physical function. The SF-12 is a self-report measure that evaluates eight domains of health, including one domain specific to physical functioning [20]. The physical functioning domain of the SF-12 associated with objective measures of lower extremity physical function, including gait speed and chair stand time [8,21]. The physical function component score was summarized with higher scores representing better physical function [22].

### Covariates

Information on covariates came from self-report or electronic medical records. Variables collected from self-report included age, weight, height, marital status, race, education, employment. Variables collected from the electronic medical record included histology of cancer, stage of cancer, time since diagnosis, cancer treatment history, weight and height at diagnosis and comorbidities (quantified using Charlson Comorbidity Index Score)[23]. BMI was calculated using weight and height (kg/m^2^). The correlation between self-reported BMI and objectively-measured BMI from medical record was 0.9775 (*P*<0.0001). But objective measures were only available on a subset sample. Therefore, we used self-reported BMI in the analysis. The Gynecologic Cancer Lymphedema Questionnaire (GCLQ) was used to assess symptoms associated with lower limb lymphedema (LLL)[16,17,24]. The GCLQ is a validated self-report measure that assesses seven domains of symptoms in both lower extremities. Participants reporting ≥ 5 symptoms of the lower extremities within the seven domains were classified as having LLL[16,17].

### Statistical Analysis

Linear regression models estimated the relationship between BMI categories and physical function (physical function component score) with 95% confidence intervals (95% CI). The *P* value for the linear trend test across categories (*P*_*trend*_) was calculated using the median value for each category as a continuous variable in the linear regression model. We examined unadjusted linear models, then adjusted for age, and subsequently built a multivariable linear model adjusting for demographic and clinical characteristics. We also adjusted LLL status (yes/no) in the multivariable linear model controlled for demographic and clinical characteristics, to assess if LLL modifies the relationship between BMI and physical function. We compared the difference in the physical function component score of our sample with U.S-population-based age-standardized normative values using the Wilcoxon rank-sum test [25]. The difference of the physical function component score was calculated using the difference in scores between participants in our sample and the age-standardized normative values of U.S general population. A dichotomized variable was generated with a 10-point lower physical function score compared to the population-based age-standardized normative values (Yes/No). A 10-point lower in physical function is clinically meaningful [3,26,27]. Multivariable linear regression model and logistic regression model were used to assess which factors associated with the difference in physical function component score and the significant lower physical function in endometrial cancer survivors. Statistical tests were two-sided, and *P* < 0.05 was the threshold for statistical significance.

## Results

### Participant Characteristics

Five hundred thirty-one participants were identified using the fellow surgical case logs and ICD-9 codes and 213 completed the mailed survey (43% response rate)[16–19]. Comparison of characteristics between women who completed the survey and who did not complete the survey showed no difference in age at diagnosis, BMI at diagnosis, and treatment modalities (S1 Table).

Demographic and clinical characteristics of the study participants are depicted in Table 1. The age of the 213 participants ranged from 29–94 years. Eighty-eight percent reported an age younger than 75 years. The majority of participants were white (83%), married or living with a partner (60%), college graduate or post-graduate degree (54%), and retired (45%). The majority of participants had endometrioid adenocarcinoma (62%) diagnosed with stage 1 disease (74%), treated with surgery (48%), were 3–4 years post diagnosis (44%), and with two or more comorbidities (61%). The BMI of study participants ranged from 14–67 kg/m^2^; 26% were normal weight (BMI<25kg/m^2^), 22% overweight (BMI:25.0–29.9kg/m^2^), 23% class I obese (BMI: 30 kg/m^2^-34.9 kg/m^2^), 14% class II obese (BMI:35kg/m^2^-39.9kg/m^2^), and 15% morbidly obese (BMI≥40.0kg/m^2^). The median of the physical function component score was 40.4 (Interquartile Range (IQR): 28.5–60.1).

**Table 1:** Demographic and Clinical Characteristics.

Variable	Total Sample (n = 213)
**Demographic Characteristics**	
Age—yr	63.6±10.6
Marital status—no. (%)	
Never married	20 (9%)
Married	128 (60%)
Divorced or separated	31 (15%)
Widowed	33 (16%)
Race—no. (%)	
White	177 (84%)
Black	28 (13%)
Other	7 (3%)
Education—no. (%)	
High school or less	46 (22%)
Some college	51 (24%)
College degree or more	114 (54%)
Employment—no. (%)	
Retired	94 (45%)
Unemployed	7 (3%)
Homemaker	16 (8%)
Other	14 (7%)
Full time	80 (38%)
**Clinical Characteristics**	
Histology type—no. (%)	
Endometrioid Adenocarcinoma	131 (62%)
Papillary serous or Clear Cell or Mixed	61 (29%)
Sarcoma	8 (4%)
Carcinosarcoma	7 (3%)
Other (Undifferentiated)	5 (2%)
Stage—no. (%)	
1	157 (74%)
2	13 (6%)
3	26 (12%)
4	8 (4%)
Unknown	9 (4%)
Treatment Modalities—no. (%)	
Surgery	101 (48%)
Surgery, Chemotherapy	18 (8%)
Surgery, Radiation	40 (19%)
Surgery, Chemotherapy, Radiation	49 (23%)
None or Unknown	4 (2%)
Time since diagnosis—no. (%)	
0–2 yrs	69 (32%)
3–4 yrs	94 (44%)
5–6 yrs	50 (23%)
BMI—kg/m^2^	31.1±8.9
Comorbidities	
0	30 (14%)
1	53 (25%)
≥2	129 (61%)

### Association between BMI Category and Physical Function

**Fig 1:**
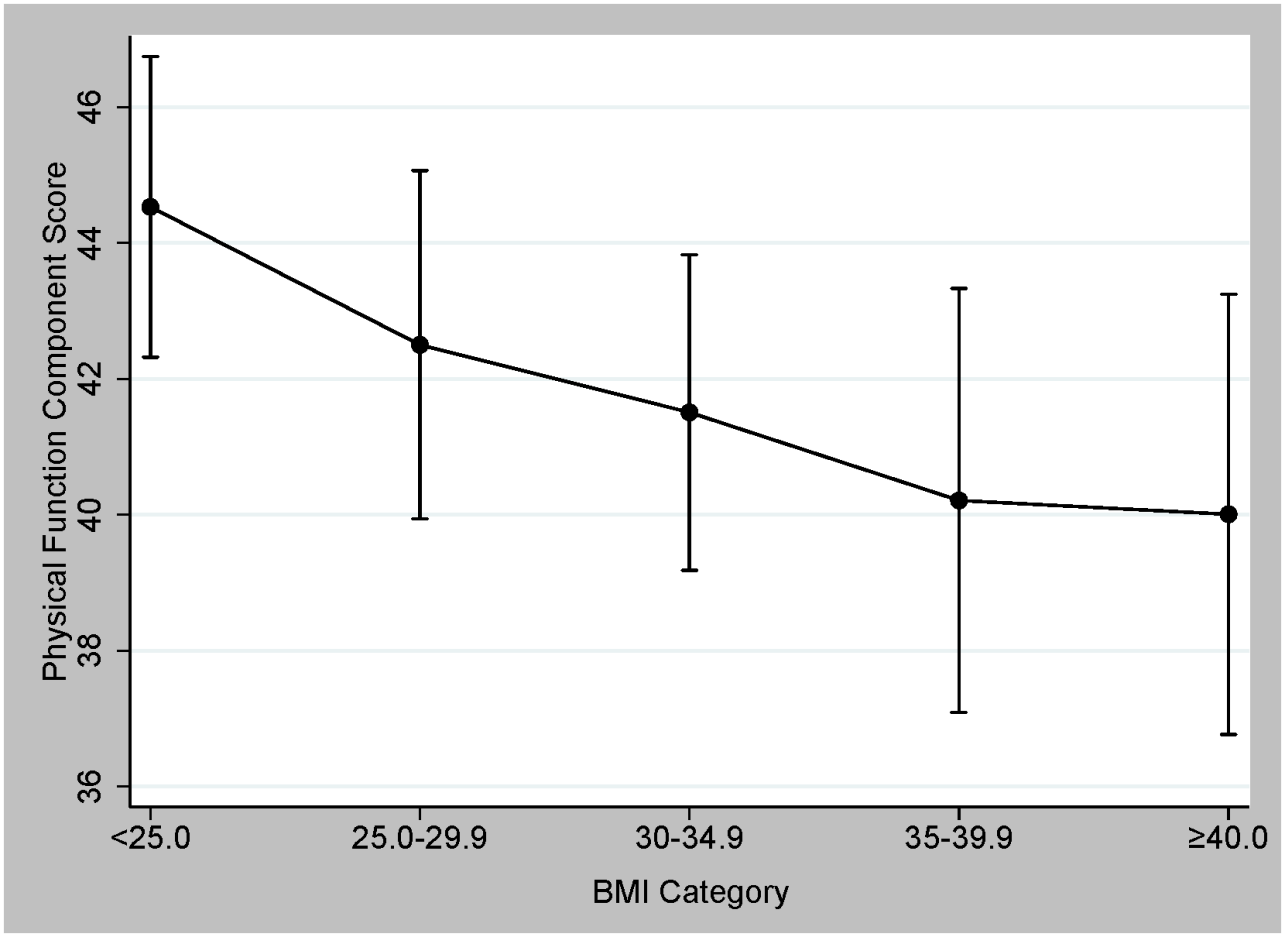
Predicted Physical Function Component Score in BMI Category.

Higher BMI categories were associated with lower physical function in all statistical models (Fig 1; *P*_*trend*_<0.004). In a multivariable-adjusted model that accounted for age, race, histology type, stage, treatment, time since diagnosis, and comorbidities, women in a higher BMI category had significantly lower physical function compared to women who were of normal weight (Table 2). When BMI was analyzed as a continuous variable, each 5-kg/m^2^ increase in BMI, physical function score was lower by 0.15 points (β = -0.15; *P* = 0.045). LLL did not modify the relationship between BMI and physical function (*P interaction* = 0.251, data not shown).

**Table 2:** Physical Function Component Score Change by BMI.

*BMI Categorical-kg/m*^*2*^	No.	Model 1^a^	P	Model 2^b^	P	Model 3^c^	P
<25	56 (26%)	0—Referent		0—Referent		0—Referent	
25.0–29.9	47 (22%)	‒2.0 (‒5.2 to 1.1)	0.209	‒2.0 (‒5.2 to 1.1)	0.209	‒1.7 (‒5.2 to 1.6)	0.311
30.0–34.9	49 (23%)	‒3.6 (‒6.6 to ‒0.6)	0.021	‒3.6 (‒6.6 to ‒0.5)	0.022	‒3.0 (‒6.3 to ‒0.2)	0.063
35.0–39.9	30 (14%)	‒4.3 (‒7.9 to ‒0.7)	0.019	‒4.3 (‒7.9 to ‒0.72)	0.019	‒4.9 (‒8.8 to ‒1.0)	0.014
≥40.0	31 (15%)	‒4.1(‒7.5 to ‒0.6)	0.022	‒4.1 (‒7.6 to ‒0.6)	0.023	‒4.6 (‒8.7 to ‒0.5)	0.027
*P*_trend_		0.005		0.005		0.003	
*BMI Continuous- kg/m*^*2*^		‒0.1 (‒0.2 to ‒0.01)	0.049	‒0.1 (‒0.2 to ‒0.01)	0.048	‒0.2 (‒0.30 to ‒0.01)	0.045

^a^Model 1 is the crude (unadjusted).

^b^Model 2 is the age adjusted.

^c^Model 3 is the fully adjusted (multivariable, controlling for age, race, education, histology type, stage, treatment, time since diagnosis, and comorbidities.

### Difference in Physical Function Component Score between Endometrial Cancer Survivors and the U.S. General Population

Compared with population-based age-standardized normative values, the physical function component score of endometrial cancer survivorssurvivors was lower among patients <75 yrs (**<45 yrs**: 39.4 vs. 52.3; **45–54 yrs**: 39.5 vs. 49.4; **55–64 yrs**: 41.1 vs. 46.9; **65–74 yrs**: 39.0 vs. 43.9, all comparisons *P*<0.05). However, among patients ≥75 years, physical function component score of our study was higher compared with the U.S general population (42.9 vs. 39.8, *P* = 0.039; Table 3). In the multivariable-adjusted linear regression, BMI was associated with differences in physical function component score between endometrial cancer survivors and the U.S general population. The physical function score was 6.8 and 6.6 lower among those with a BMI of 35–39.9kg/m^2^ (*P* = 0.002) and BMI ≥40kg/m^2^ (*P* = 0.004) compared with BMI<25kg/m^2^ (Table 4). When BMI was analyzed as a continuous variable, higher BMI associated with lower physical function score (*β* = -0.27; *P*_trend_ = 0.001). Among patients <75 yrs, compared to those with a BMI<25kg/m^2^, the odds of a physical function score 10 points lower than the general population was 6.0 in those with a BMI of 35–39.9kg/m^2^ (*P* = 0.007) and 4.0 in those with a BMI≥ 40kg/m^2^ (*P* = 0.046). When BMI was analyzed as a continuous variable, higher BMI was associated with an increased odds of a 10-point lower physical function score compared with the general population (OR = 1.06; *P*_*trend*_ = 0.018). We also explored BMI in a non-linear (quadratic) relationship, but this did not reach the threshold of statistical significance (*P* = 0.083).

**Table 3:** Difference in Physical Function Component Score between Endometrial Cancer Survivors and the U.S. General Population.

Age Categories	Sample size	Physical function component score (Median)			
		Difference	Endometrial cancer survivors	The U.S general population	P value
Over all	213(100%)	-3.1	42.1	45.2	<0.001
<45	13 (6%)	-12.9	39.4	52.3	0.0096
45–54	22 (10%)	-9.9	39.5	49.4	0.0007
55–64	80 (38%)	-5.8	41.1	46.9	<0.001
65–74	71 (33%)	-4.9	39.0	43.9	0.0019
≥75	26 (12%)	3.1	42.9	39.8	0.0390

**Table 4:** Linear Regression Model to Assess the Difference of Physical Function Component Score between Endometrial Cancer Survivors and The U.S General Population.

	Coef.	P value	95% CI	
BMI Categorical				
<25 *kg/m*^*2*^	Ref	—	—	—
25.0–29.9 *kg/m*^*2*^	-2.22	0.238	-5.91	1.48
30.0–34.9 *kg/m*^*2*^	-3.23	0.068	-6.71	0.25
35.0–39.9 *kg/m*^*2*^	-6.79	0.002	-11.01	-2.56
≥40.0 *kg/m*^*2*^	-6.62	0.004	-11.04	-2.20
Race				
white	Ref	—	—	—
black	9.69	0.128	-2.81	22.19
other	1.45	0.469	-2.49	5.38
unknown	-2.00	0.616	-9.86	5.86
Education				
High school or less	Ref	—	—	—
Some college	2.58	0.176	-1.17	6.33
College degree or more	1.65	0.331	-1.69	4.99
Stage				
1	Ref	—	—	—
2	4.24	0.134	-1.32	9.80
3	-0.45	0.838	-4.80	3.90
4	0.63	0.878	-7.48	8.75
Histology type				
Endometroid Adenocarcinoma	Ref	—	—	—
Papillary serous or Clear Cell or Mixed	-0.08	0.959	-3.28	3.11
Sarcoma	-2.91	0.453	-10.54	4.72
Carcinosarcoma	0.17	0.960	-6.47	6.81
Other (Undifferentiated)	-6.74	0.188	-16.81	3.32
Treatment				
Surgery	Ref	—	—	—
Surgery, Chemotherapy	-3.39	0.192	-8.50	1.72
Surgery, Radiation	-1.81	0.330	-5.46	1.84
Surgery, Chemotherapy, Radiation	2.53	0.228	-1.60	6.66
Years from Diagnosis	-0.67	0.18	-1.65	0.31
Charlson Comorbidity				
0	Ref	—	—	—
1	0.84	0.684	-3.22	4.90
≥2	1.32	0.481	-2.36	5.00

## Discussion

Our findings indicated that endometrial cancer survivors with a higher BMI have lower physical function compared with those of normal weight. Previous studies estimated that 35% of endometrial cancer survivors report poor physical function [16], and 53% experienced one or more physical function impairments [18]. LLL is the most common physical function impairments and associated with poorer physical function [16,18]. Physical function predicts mortality and major health outcomes (disability, death and hospitalization) in the general population as well as cancer survivors [5–9]. Obesity is one of the barriers to the survivorship in endometrial cancer population [28], which related to poorer physical function [12,13,29,30], and associated with disability [31]. Higher BMI increases overall and disease-specific mortality in endometrial cancer survivors [32]. In this study, 52% of participants were obese. Obesity could be one of the main contributors to poor physical functioning and diminished long-term quality of life in endometrial cancer survivors.

Interestingly, our data indicate that endometrial cancer survivors who were <75yrs report lower physical function compared with the U.S general population. Conversely, those ≥75yrs report better physical function. One explanation would be those who were diagnosed with endometrial cancer at a younger age were suffering more health issues, such as obesity and other comorbidities, compared with the general population, while those who were diagnosed with endometrial cancer at an age of 75 or older were less likely to have any more health issues compared with age-standardized women without endometrial cancer. It could also result from potential response bias that younger endometrial cancer survivors may be more likely to report how sicker they were, whereas older patients may be more likely to report how well they were living with cancer. This hypothesis-generating observation warrants addition investigation.

Health concerns of cancer survivors have included physical function, in addition to surviving from cancer. Studies have shown that cancer survivors are more likely to report worse physical function compared with healthy populations [14,15,33]. Our study found that BMI is the only factor associated with differences in physical function between endometrial cancer survivors and the U.S general population. Compared to the cancer-free population, the physical function of cancer survivors’ declines with an accelerated trajectory after cancer diagnosis, and obesity predicts functional decline [34]. Although some of the deleterious conditions resulting from endometrial cancer and treatment are not reversible, obesity and physical function could be improved by lifestyle interventions. Exercise can improve physical function in cancer patients as well as obese frail older adults [35,36]. Slowly progressive weight lifting program in the breast cancer population prevented the deterioration of self-reported physical function [3]. Weightlifting appears safe even in women with lower extremity lymphedema [37]. Other lifestyle intervention studies also suggest that exercise could prevent physical function decline in the older population, and a combination of weight loss and exercise can provide greater improvement in physical function [38,39].

We acknowledge that underestimates of body size and BMI are well documented, especially in obese population [40,41]. In our experience in gynecologic practices in our institution, most of obese endometrial cancer survivors have little awareness and concerns about their weight. Recommendations from health care providers could significantly increase physical activity [42,43]. And patients may be more likely to follow the clinicians’ recommendation given the perception that cancer is more life-threatening than other diseases [44,45]. Given the large proportion of endometrial cancer survivors who are overweight and/or obese, and have other health-issues that need medically-based supervised exercise program [28], health care providers may play an important role to increase patients' awareness of weight and body size, and refer them to a medically-based lifestyle intervention, to reduce body weight, improve overall body composition, and increase physical activity levels to yield improvements in a variety of clinical and patient-reported outcomes in endometrial cancer population. Several behavior interventions focused on diet and exercise also showed the effectiveness of weight loss in endometrial cancer survivors [44,45].

The major limitation of this study is the cross-sectional study design, which does not allow us to clarify the temporal relationship of the variables in our analysis. We were unable to integrate objective measures of physical function, which may provide complementary information to self-reported measures. Nonetheless, self-reported measures of physical function are clinically meaningful [8,21]. A prospective designed study using objective measurements of physical function could help to determine the causal relationship and predictive factors of poor physical function in endometrial cancer. In addition, our study was conducted within a single health system. Characteristics of endometrial cancer patients seen within the academic health system may differ from the community setting. For example, 80% of our study sample was diagnosed at an early stage, and 88% were younger than 75 yrs old, which may not represent the general endometrial cancer population. Furthermore, women who chose to participate in the study differ from those who did not participate. Survey participants were more often diagnosed with earlier stage cancer, suggesting a possible selection bias. However age and BMI did not differ between survey participants and non-participants. Although we found physical function in our sample is quite different compared with the U.S age-standardized population, it would be helpful to have BMI-adjusted normative values to compare the relationship. Further prospective studies with larger samples and additional measures of physical function are needed to provide continued guidance on the preservation of physical function in this population.

## Conclusion

Among endometrial cancer survivors, higher BMI is associated with lower physical function. Younger endometrial cancer survivors (<75yrs) report lower physical function compared to age-standardized normative values. If these cross-sectional findings reflect a causal relationship, this would suggest that referring younger obese endometrial cancer survivors to a lifestyle intervention targeting in weight loss, such as diet and exercise, may benefit them to maintain and improve physical function in terms to help their recovery from cancer and related treatment. The goal of these interventions would be to improve their ability to perform daily activity and to live independently. Additional investigation is warranted to identify appropriate approach to refer endometrial cancer survivors into effective lifestyle intervention to improve their quality of life.

## Supporting Information

S1 File De-identified Data for Sharing (As Requested). (DTA)

S1 Table Comparison of Characteristics between Endometrial Cancer Survivors Who Completed Survey and Who Didn’t Complete Survey. (DOCX)
