# Supplementary material for: Association between Body Mass Index and Physical Function among Endometrial Cancer Survivors
Source: PLoS One. 2016 Aug 16;11(8):e0160954. doi: 10.1371/journal.pone.0160954 (PMC4986945; doi:10.1371/journal.pone.0160954)
Supplement: S1 Table — (DOCX) [file pone.0160954.s002.docx]

S1 Table. Comparison of Characteristics between Endometrial Cancer Survivors who completed survey and who didn’t complete survey

| Variable | | Survey Completed (n=213) | Survey Not Completed (n=273) | P value |
| --- | --- | --- | --- | --- |
| **Demographic Characteristics** | |  |  |  |
|  | Age at diagnosis — yr | 60.1±10.6 | 60.1±11.4 | 0.45 |
|  | Race — no. (%) |  |  | 0.002 |
|  | White | 177 (84%) | 180 (66%) |  |
|  | Black | 28 (13%) | 64 (23%) |  |
|  | Other | 7 (3%) | 29 (11%) |  |
| **Clinical Characteristics** | |  |  |  |
|  | Stage — no. (%) |  |  | 0.015 |
|  | 1 | 157 (74%) | 176 (64%) |  |
|  | 2 | 13 (6%) | 24 (9%) |  |
|  | 3 | 26 (12%) | 39 (14%) |  |
|  | 4 | 8 (4%) | 14 (5%) |  |
|  | Unknown | 9 (4%) | 20 (7%) |  |
|  | Treatment Modalities — no. (%) |  |  | 0.524 |
|  | Surgery | 101 (48%) | 136 (50%) |  |
|  | Surgery, Chemotherapy | 18 (8%) | 26 (9%) |  |
|  | Surgery, Radiation | 40 (19%) | 54 (20%) |  |
|  | Surgery, Chemotherapy, Radiation | 49 (23%) | 51 (19%) |  |
|  | None or Unknown | 4 (2%) | 6 (2%) |  |
|  | BMI at diagnosis — kg/m^2^ | 32.1±9.3 | 32.3±9.1 | 0.81 |

*total n=479. 7 of those who completed survey were excluded from the EMR due to lack of follow up info.
